# Prevalence of endoparasites by microscopic analysis in free-range chickens in a Brazilian semiarid region

**DOI:** 10.1590/S1984-29612022063

**Published:** 2022-12-12

**Authors:** Juliana Trajano da Silva, Felipe Boniedj Ventura Alvares, Clarisse Silva de Menezes Oliveira, Luana Carneiro de Sousa, Brendo Andrade Lima, Thais Ferreira Feitosa, Arthur Willian de Lima Brasil, Vinícius Longo Ribeiro Vilela

**Affiliations:** 1 Programa de Pós-Graduação em Ciência Animal, Universidade Federal de Campina Grande – UFCG, Patos, PB, Brasil; 2 Departamento de Medicina Veterinária, Instituto Federal da Paraíba – IFPB, Sousa, PB, Brasil; 3 Departamento de Morfologia, Universidade Federal da Paraíba – UFPB, João Pessoa, PB, Brasil

**Keywords:** Poultry, *Eimeria* spp., *Trichuris* spp., superfamily Heterakoidea, Avicultura, *Eimeria* spp., *Trichuris* spp., superfamília Heterakoidea

## Abstract

The aim of this study was to evaluate the prevalence and diversity of endoparasitic fauna and the risk factors associated with parasite infections in free-range chickens in the state of Paraíba, northeastern Brazil. Ten municipalities were visited and, in each of them, ten farms, to collect animal feces and apply epidemiological questionnaires. Feces from 417 poultry were used to perform EPG (eggs per gram) and OoPG (oocysts per gram) tests. Prevalences of 40.52% (169/417) and 39.08% (163/417) were observed for nematodes and coccidia, respectively. In 17% (71/417), mixed infections by nematodes and coccidia were observed. Nematodes of Heterakoidea superfamily were present in 100% of the positive samples (169/169), followed by *Trichuris* spp. (57.3%; 97/169). All the protozoan oocysts observed belonged to the genus *Eimeria* (100%; 163/163). The variable of presence of drooping wings was considered to be a factor associated with infection by coccidia (odds ratio = 5.412; confidence interval: 1.179-24.848; p = 0.030). It was concluded that there is high prevalence of nematodes and coccidia in free-range chickens in the state of Paraíba, Brazil. Better sanitary management measures, with greater hygiene of facilities, together with chemical control of parasites, can improve productivity by reducing the rate of gastrointestinal parasites.

## Resumo

O objetivo do estudo foi avaliar a prevalência e diversidade da fauna endoparasitária, e os fatores de risco associados às infecções em galinhas de fundo de quintal no Estado da Paraíba, Nordeste do Brasil. Dez municípios foram visitados e, em cada um, dez fazendas, para coletar fezes e aplicar um questionário epidemiológico. Fezes de 417 aves foram utilizadas para a realização de testes de OPG (ovos por grama) e OoPG (oocistos por grama). Prevalências de 40,52% (169/417) e 39,08% (163/417) foram observadas para nematoides e coccídios, respectivamente. Em 17% (71/417), infecções mistas por nematoides e coccídios foram observadas. Nematoides da superfamília Heterakoidea estavam presentes em 100% das amostras positivas (169/169), seguidos por *Trichuris* spp. (57,3%; 97/169). Todos os oocistos de protozoários observados pertenceram ao gênero *Eimeria* (100%; 163/163). A variável de presença de asas caídas foi considerada como fator associado à infecção por coccídios (odds ratio= 5,412; interval de confiança: 1,179-24,848; p = 0,030). Concluiu-se que é alta a prevalência de nematoides e coccídios em galinhas de fundo de quintal no Estado da Paraíba. Melhorias nas medidas de manejo sanitário, com maior higiene das instalações, associados a um controle químico de parasitos, podem melhorar a produtividade pela redução das taxas de parasitismo gastrointestinal.

## Introduction

Poultry farming is one of the pillars of the Brazilian economy. Brazil is the second largest producer of chicken meat in the world, with most of this production destined for domestic consumption. Production of free-range chickens (*Gallus gallus domesticus*) or backyard chickens in Brazil is undertaken on almost all farms, and this forms part of the subsistence resources of most small-scale farmers (Sobral et al., 2010).

Rearing of free-range chickens on rural family smallholdings is distinguished by the idea of ​​extensive management. However, facilities and practices for efficiently favoring reproductive, nutritional and sanitary conditions are often precarious. The free-range poultry industry is governed by the breeding conditions specified in circular letter number 007/99, dated May 19, 1999, from the Division of Industrial Operations/Department of Inspection of Products of Animal Origin (DOI/DIPOA) (Brasil, 2012). This sets forth the conditions required for feeding, handling, age at slaughter and lineage. It specifies that chickenfeed should consist of ingredients, including proteins, that are exclusively of plant origin. It states that management should consist of keeping the young chicks in sheds for up to 25 days and that they should then be released into a field, such that extensive rearing is practiced from then on, with at least three-square meters of pasture per poultry. The age at slaughter should be at least 85 days. Regarding lineage, the directive states that only breeds suitable for this purpose should be used.

The aim of extensive production systems is to promote natural behaviors and increase animal welfare. However, these systems are characterized by high levels of helminth infections (Wuthijaree et al., 2017).

In extensive exploitation of chickens, the prevalence of parasitic infections is around 50% in flocks. This has an impact in terms of economic losses, such as higher mortality rate and higher costs of prophylaxis. Knowledge of the epidemiology of parasites and their impacts on production units is extremely important. Only through this can control and prophylaxis measures for these diseases be encouraged (Sharma et al., 2019).

Helminthiasis and coccidiosis of domestic poultry are most often asymptomatic. However, they can cause nutritional deficiencies, predispose to secondary infections, interfere with post-vaccination immune development and lead to death (Guerra et al., 2015; Silva et al., 2016).

The most frequently reported gastrointestinal helminths parasitizing domestic poultry are from the superfamily Heterakoidea, composed of the genera *Ascaridia* spp. and *Heterakis* spp., which belong to the phylum Nematoda (Taylor et al., 2016). In cases of high levels of infection, the pathogenicity of these parasites is also higher (Thapa et al., 2015; Silva et al., 2018).

Coccidian infections are highly prevalent among free-range poultry worldwide. These infections cause significant economic losses through high morbidity and mortality (Blake & Tomley, 2014). The main coccidia of domestic chickens are obligate intracellular protozoa of the genus *Eimeria*, with seven different species: *Eimeria acervulina*, *Eimeria brunetti*, *Eimeria maxima*, *Eimeria mitis*, *Eimeria necatrix*, *Eimeria praecox* and *Eimeria tenella*. The species that usually cause the greatest economic losses are *E. tenella*, *E. maxima*, *E. necatrix* and *E. brunetti* (Kaboudi et al., 2016).

In the southern and southeastern regions of Brazil, the vast majority of chickens are parasitized by nematodes and coccidia. The main parasites are: *Capillaria* sp., *Ascaridia galli*, *Heterakis gallinarum* and *Eimeria* spp. (Thapa et al., 2015; Abebe & Gugsa, 2018). In the northeastern region, there are few studies on the occurrence and severity of infection by endoparasites in free-range chickens, which may vary due to geoclimatic conditions (Gomes, 2009; Silva et al., 2016, 2018).

Thus, we aimed to evaluate the endoparasitic fauna of chickens, detail their prevalence and factors associated with infections and estimate their economic importance in subsistence farming, in an extensive system in the state of Paraíba, northeastern Brazil.

## Material and Methods

### Research locations

This study was conducted in the Sertão mesoregion of the state of Paraíba, northeastern Brazil, between February and August 2021. The area has a semiarid climate, with average rainfall of 250 to 800 mm. The rains are irregular, concentrated mainly between the months of March and May. It has a maximum temperature of 32 ºC and a minimum of 20 ºC, with high evaporation rates and relative air humidity close to 70% (INMET, 2010). The Sertão mesoregion is made up of 83 municipalities, covering an area of 56,467,242 km^2^, equivalent to 40% of the state territory (IBGE, 2009).

### Sample population

The sample design used was a cross-sectional study and the sampling was designed to determine the prevalence of positive farms (foci). The study was conducted in two stages: (1) a random selection of a pre-established number of farms (primary units); and (2) within the primary units, a pre-established number of free-range chickens (secondary units) were randomly sampled.

Sampling was performed in two stages. Initially, farms were selected through simple random sampling, as recommended by Thrusfield (2007):


$$n=\frac{{\text{z}}^{2}.\text{P}\left(1-\text{P}\right)}{{\text{d}}^{2}}$$ (1)

Where:

n: number of selected farms;

z: Value of the normal distribution at the confidence level

P: expected prevalence;

d: margin of error .

The sampling of farms was performed at a confidence level of 95%, expected prevalence of 50% and margin of error of 10%.

This was adjusted for the local population through the formula:


$$n_{ajus}=\;\frac{\text{N}.\text{n}}{\text{N}+\text{n}}$$ (2)

Where:

n_ajus_: final number of farms selected;

n: number of farms selected;

N: number of farms that exist.

The second stage was to determine the number of poultry per farm based on detection of the disease in the flock, as described by Thrusfield (2007):


$$n_{ani}=\;\left[1-\;\left(1-{\text{p}}^{\frac{1}{\text{d}}}\right)\right]\;.\;\left(\text{N}-\;\frac{\text{d}}{2}\right)+1$$ (3)

Where:

n_ani_: sample size required;

N: size of the population on the farm;

d: number of poultry affected in the population;

p: Probability of finding at least one case in the sample.

**Figure 1:**
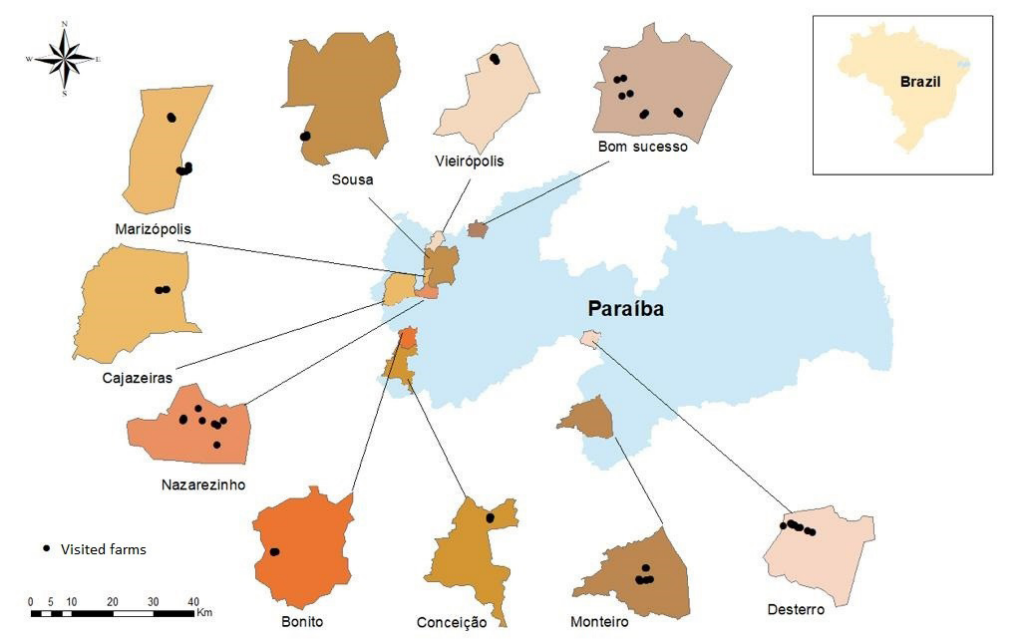
Geographical locations of free-range chicken farms evaluated for the presence of infection by nematodes and coccidia in the semiarid region of Paraíba, Brazil.

In the mesoregion studied, there are 99,545 chicken-producing farms (IBGE, 2017), and 96 of these (10% error) were needed to make up the sample. However, for safety reasons, samples were collected from 100 farms. The geographical locations of the farms visited are described in Figure 1.

After determining the minimum number of farms, it was necessary to calculate the number of animals to be evaluated in each farm. Thrusfield (2007) recommends estimating the probability of having at least one infected person based in the number of animals and an expected prevalence based on previous studies. Therefore, an expected prevalence of 50% was adopted according to Sharma et al. (2019) and the number of samples was determined: farms that had up to 30 poultry, four samples were randomly selected; and on those that had more than 30 poultry, five were selected. On farms that only had up to four poultry, samples were collected from all of them. This analysis was also performed at the 95% confidence level.

### Collection of samples for parasitological analysis

Feces samples were collected directly from the rectal ampulla of the poultry, during the breeding and fattening phases, regardless of sex or breed, during the visits to the farms. After collection, the material was sent to the Veterinary Parasitology Laboratory of the Federal Institute of Education, Science and Technology of Paraíba, Sousa Campus. From these fecal samples, floatation methods of eggs per gram (EPG) of feces and oocysts per gram (OPG) of feces were individually made in order to determine the levels of infection by nematodes and coccidia, respectively (Gordon & Whitlock, 1939).

In the case of samples that were positive for coccidia, an aqueous solution of 2.5% potassium dichromate (K_2_Cr_2_O_7_) was added to the containers, such that the volumetric proportions were 16.7% feces and 83.3% potassium dichromate solution. These samples were then kept in a BOD incubator at an average temperature of 28ºC for 15 days, for sporulation of the oocysts.

### Data collection

On the farms visited, structured epidemiological questionnaires were applied to collect information about variables that may act as possible factors associated with infections. The variables investigated were the following: age group, breed reared, breeding system, type of farm, poultry management, farm area, number of animals, clinical signs observed, anthelmintic used, animal deworming protocol, egg production evaluation, mortality rate and disease occurrence and prevention.

### Statistical analysis

Data obtained from the questionnaires were statistically analyzed in two stages (univariate analysis and multivariate analysis), to determine associations between epidemiological and clinical variables and the results from parasitological examinations for nematode and coccidian infections. In the univariate analysis, the groups were compared against the independent variables and their respective categories. Independent variables that presented p-values ≤ 0.2 through the chi-square test or Fisher's exact test were selected for multivariate analysis, using multiple logistic regression (Hosmer & Lemeshow, 2000). The fit of the final model was verified using the Hosmer and Lemeshow test, whereby a p-value ≥ 0.5 indicated a satisfactory fit; and using the coefficient of determination (R^2^). The significance level adopted was 5%, and the analyses were performed using the SPSS 23.0 for Windows software.

## Results

The total number of poultry samples collected for EPG and OoPG tests was 417, of which 62.6% (261) were found to be infected by some gastrointestinal parasites. The prevalence of nematode infections was 40.5% (169/417) and coccidia, 39.1% (163/417). In 17% (71/417) of the samples, there was infection by both nematodes and coccidia. In 90% of the 100 farms evaluated, endoparasitism was observed in at least one poultry.

The most prevalent nematodes were from the superfamily Heterakoidea, which was present in 100% of the positive samples (169/169); followed by *Trichuris* spp., in 57.3% (97/169). All the protozoan oocysts observed in the samples belonged to the genus *Eimeria* (100%; 163/163).

Different categories were established for assessing the levels of parasite loads among the poultry (Table 1). Regarding nematodes and coccidia, the average level was the most prevalent (EPG: > 500 and ≤ 3000; OoPG: >1000 and ≤ 5000).

**Table 1:** Level of infection by nematodes and *Eimeria* spp. in free-range chickens in the state of Paraíba, Brazil.

EPG^1^	Nematodes		OoPG^2^	*Eimeria*	
	Total	%		Total	%
Low (≤ 500)	39	23.0	Low (≤ 1,000)	45	27.6
Medium (501-3,000)	72	42.7	Medium (1,001-5,000)	89	54.6
High (> 3,001)	58	34.3	High (> 5,001)	29	17.8
Total	169	100.0	Total	163	100.0

1 Eggs /g;

2 oocysts/g.

Among the variables analyzed in the epidemiological questionnaires, those that presented P ≤ 0.2 for infections by nematodes and coccidia in the univariate analysis are described in Tables 2 and 3, respectively. Although, in the multivariate analysis, using multiple logistic regression, there were no factors associated with nematode infection and only the variable of presence of drooping wings was considered to be a factor associated with infection by coccidia (odds ratio = 5.412; confidence interval: 1.179-24.848; p= 0.030). Production of free-range chickens without a defined breed pattern was observed in all farms (100%; 100/100). This production was a way for small-scale farmers to supplement their income on 42% (42/100) of the farms. Among these, 15% (15/100) sold eggs, 16% (16/100) sold live or slaughtered poultry and 11% (11/100) sold chicks. Among the farms from which poultry were sold, chicken production was the focus and main source of income for only 2% (2/100). On 58% (58/100) of the farms, the focus of rearing chickens was on consumption of these chickens and their byproducts by the farming family.

**Table 2:** Univariate analysis on factors associated with nematode positivity on farms producing free-range chickens in the state of Paraíba, Brazil.

Variable		farms (n)	+ farms (%)	p
Balanced feed				0.128
	No	60	47 (78.3)	
	Yes	40	36 (90.0)	
Birth rate				0.159
	1 - 30	83	71 (85.5)	
	> 30	17	12 (70.6)	
Diarrhea				0.110
	No	77	61 (79.2)	
	Yes	23	22 (95.7)	
Pallor				0.182
	No	81	65 (80.2)	
	Yes	19	18 (94.7)	

**Table 3:** Univariate analysis on factors associated with positivity for coccidia on farms producing free-range chickens in the state of Paraíba, Brazil.

Variable		farms (n)	+ farms (%)	p
Cleaning of facilities				0.050
	Yes	51	34 (66.6)	
	No	49	41 (83.7)	
Cleaning of feeders				0.127
	Yes	41	34 (82.9)	
	No	59	41 (69.5)	
Cleaning of drinkers				0.127
	Yes	50	40 (80)	
	No	50	35 (70)	
Feed				0.128
	Corn	41	33 (80.5)	
	Leftover food	37	30 (81.1)	
	Balanced feed	40	31 (77.5)	
	Mixed	13	11 (84.6)	
Feeding container				0.029
	Bowl	64	47 (73.4)	
	Floor	34	28 (82.3)	
	Feeder	2	0 (0.0)	
Weather when most sick				0.040
	Rainy	72	50 (69.4)	
	Dry	28	25 (89.3)	
Rearing system				0.174
	Extensive	79	56 (70.8)	
	Intensive	19	17 (89.5)	
	Semi-intensive	2	2 (100)	

Rudimentary installations were observed on almost all the farms. The feeding place for the poultry consisted of ordinary bowls on 65 farms, while the feed for the poultry was strewn on the ground on 33 farms and the feed for the poultry was placed in appropriate feeders on only two farms. The water supplied to drinking fountains consisted of treated water on only four farms, while it was cistern water on 58 farms and it came from a weir on 38 farms. The facilities on most of the farms (63 of them) had earth floors, and while only 37 had cemented floors for the poultry.

## Discussion

The high prevalence of gastrointestinal parasites observed among the chickens of this study (62.6%), together with the high percentage of the farms that had parasitized poultry (90%), reflected errors in sanitary management on most of the farms evaluated, such as non-cleaning of facilities (p=0.05), which may have favored parasite transmission. According to Thapa et al. (2017), factors such as temperature, poultry lineage and different environmental conditions can influence the development and maintenance of parasites on farms.

The high prevalence of nematodes (40.52%; 169/417) was similar to what had previously been described in the state of Maranhão, Brazil, of 32.6%, with predominance of nematodes from the superfamily Heterakoidea (75.6%) (Saraiva et al., 2021). Van et al. (2020), who evaluated parasitosis among backyard chickens in Vietnam, found a prevalence of nematodes of 75%, among these poultry. In turn, Thapa et al. (2015), who evaluated gastrointestinal parasites in chickens that were slaughtered from an organic farming system in eight European countries, found that the prevalence was 69.5%.

During the direct search for parasite eggs in coprological examinations on chickens, it was not possible to visually differentiate the species *H. gallinarum* and *A. galli*, as they have very similar eggs. These are characterized only as belonging to the superfamily Heterakoidea (Wuthijaree et al., 2017; McDougald, 2020).

The nematode disease caused by *A. galli* and *H. gallinarum* in association is very common, with parasitism in different areas of the intestinal tract: *A. galli* parasitizes the small intestine and *H. gallinarum*, the cecum. Both the adult and larval stages of *A. galli* cause destruction of the intestinal epithelium and mucosal necrosis (Ferdushy et al., 2016). *H. gallinarum* can, in addition to causing weight loss, be a carrier of the protozoan *Histomonas meleagridis*, which causes histomonosis, a disease with high morbidity and lethality (Brener et al., 2006). Once these parasites settle in, they are difficult to control, requiring chemical treatments and strict sanitary measures, with no guarantee of success (Uhuo et al., 2013).

For coccidia, the observed prevalence was 39.1%. This differed from what was found by Toledo et al. (2011), in the northern region of Brazil (63.1%), and by Moraes et al. (2015), in the southern region of Brazil (96%). This difference was probably due to the climatic conditions of those regions, which differ from the semiarid region of northeastern Brazil, where rainfall is concentrated in a few months and there is low humidity throughout the year. These, plus the high temperatures of the latter region, can considerably reduce the survival rate of oocysts in the environment and mainly to make the sporulation process unfeasible.

The direct losses caused by avian coccidiosis include lower weight gain, increased mortality, increased secondary infections and the costs of chemotherapy treatments. There are also indirect costs, related to use of drugs and/or vaccines to prevent infections, consequent to failed immunization due to the lack of specific antibodies for each species (Blake & Tomley, 2014).

Among the positive animals, there was a predominance of medium-level infection due to nematodes and coccidia. The monetary losses due to parasitic infections are believed to be high, although they are difficult to quantify. Aguiar & Tavernari (2010) reported that this type of infection can increase susceptibility to other endemic pathogens, affecting animals through reduced food intake, low weight gain and reduced feed conversion.

Infection by gastrointestinal nematodes and coccidia, even if subclinical, causes low feed conversion. Therefore, losses occur with regard to feeding, and this also predisposes to development of diseases caused by opportunistic agents (Shirzad et al., 2011).

Infection by multiple species of gastrointestinal nematodes is common. However, infections with EPG values ​​up to 3000 are believed not to affect the growth performance of chickens (Wuthijaree et al., 2018). The average levels of infections and their subclinical characteristics are extremely important, given that the parasites continue to reproduce and release eggs and oocysts into the environment, thus causing more animals to become infected or intensification of the disease in more susceptible poultry.

For *Eimeria* spp. infections, the dry weather was significant (p=0.040) for the variable weather when most sick. In the Brazilian semi-arid, dry weather occurs between July to December, with high mean temperatures over 26 ºC, and maximum temperatures over 32 ºC (INMET, 2010). Chicken submitted to heat stress reduces growth and egg production all over the world (Biswal et al., 2022). The immune organs of chicks at different ages submitted to heat stress of displayed significant atrophy and edema, necrosis of bursa cells and reduced both cell-mediated and humoral immunity (Li et al., 2015). Broilers that were subjected to heat stress showed decreased immunity, and they became much more susceptible to pathogens, such as coccidia (Lara & Rostagno, 2013; Calefi et al. 2014; Vandana et al., 2021).

It was observed that on the farms where feed was simply strewn on the ground, the chickens were more commonly positive for coccidiosis (82.4%) than were those whose feed was presented in feeders or bowls (74.2%) (Table 3). However, poultry that are reared in backyards still have contact with the environment and perform natural behaviors, such as scratching and looking for small insects. Such behaviors make these animals more exposed to parasitic infection (Ferdushy et al., 2016; Wuthijaree et al., 2017; Bawm et al., 2021).

The sign of drooping wings was considered to be a factor associated with coccidian infection (odds ratio = 5.412). This parasitosis normally follows a course that involves diarrhea, reduced food absorption capacity and consequent weight loss (Taylor et al., 2016). Malnutrition and reduced ability to absorb food make the poultry appear asthenic and, thus, they fail to keep their wings in an anatomical position, which results in the final symptom of drooping wings.

Management is the main way to avoid economic losses from helminths and coccidiosis, including hygiene, biosecurity and environmental management practices (Abebe & Gugsa, 2018). Rudimentary installations that make cleaning difficult, poor quality of water offered to chickens and feeding them by strewing the feed on the ground are factors that may explain the high rates of infections in the poultry evaluated. Production of free-range chickens was a form of income supplementation on almost half of the farms visited, which were suffering economic losses due to the lack of correct parasite control among their chickens.

The semiarid region of Paraíba has a natural advantage for controlling these diseases, due to its high temperatures and low humidity, which, according to Thapa et al. (2017), reduce the survival of eggs and oocysts of the parasites in the environment. Therefore, by combining this favorable condition for parasite control with correct sanitary management, through incentive programs aimed at improving facilities and correct treatment of animals, better infection control can be obtained. In this manner, greater animal welfare and greater productivity, whether eggs, chicks or carcasses, can be envisaged.

## Conclusion

There is a high prevalence of nematodes and coccidia in free-range chickens in Paraíba, Brazil. The most prevalent nematodes were found to be those of the superfamily Heterakoidea, and all the coccidia were from the genus *Eimeria*. The clinical sign of drooping wings was considered to be a factor associated with infection by coccidia, and it can be assumed that this is due to the pathophysiology of the disease. Incentives to improve facilities and sanitary management, with greater hygiene rigor, avoiding heat stress, along with chemical control of parasites, can improve productivity by reducing the rate of occurrence of gastrointestinal parasites on farms.
